# Deep Learning-driven research for drug discovery: Tackling Malaria

**DOI:** 10.1371/journal.pcbi.1007025

**Published:** 2020-02-18

**Authors:** Bruno J. Neves, Rodolpho C. Braga, Vinicius M. Alves, Marília N. N. Lima, Gustavo C. Cassiano, Eugene N. Muratov, Fabio T. M. Costa, Carolina Horta Andrade

**Affiliations:** 1 Laboratory of Cheminformatics, University Center of Anápolis – UniEVANGÉLICA, Anápolis, Goiás, Brazil; 2 LabMol – Laboratory for Molecular Modeling and Drug Design, Faculty of Pharmacy, Federal University of Goiás, Goiânia, Goiás, Brazil; 3 InsilicAll, São Paulo, São Paulo, Brazil; 4 Laboratory for Molecular Modeling, UNC Eshelman School of Pharmacy, University of North Carolina, Chapel Hill, North Carolina, United States of America; 5 Laboratory of Tropical Diseases–Prof. Dr. Luiz Jacintho da Silva, Department of Genetics, Evolution, Microbiology and Immunology, Institute of Biology, University of Campinas, Campinas, São Paulo, Brazil; 6 Global Health and Tropical Medicine (GHTM), Instituto de Higiene e Medicina Tropical (IHMT), Universidade Nova de Lisboa (UNL), Lisboa, Portugal; 7 Department of Chemical Technology, Odessa National Polytechnic University, Odessa, Ukraine; University of Toronto, CANADA

## Abstract

Malaria is an infectious disease that affects over 216 million people worldwide, killing over 445,000 patients annually. Due to the constant emergence of parasitic resistance to the current antimalarial drugs, the discovery of new drug candidates is a major global health priority. Aiming to make the drug discovery processes faster and less expensive, we developed binary and continuous Quantitative Structure-Activity Relationships (QSAR) models implementing deep learning for predicting antiplasmodial activity and cytotoxicity of untested compounds. Then, we applied the best models for a virtual screening of a large database of chemical compounds. The top computational predictions were evaluated experimentally against asexual blood stages of both sensitive and multi-drug-resistant *Plasmodium falciparum* strains. Among them, two compounds, LabMol-149 and LabMol-152, showed potent antiplasmodial activity at low nanomolar concentrations (EC_50_ <500 nM) and low cytotoxicity in mammalian cells. Therefore, the computational approach employing deep learning developed here allowed us to discover two new families of potential next generation antimalarial agents, which are in compliance with the guidelines and criteria for antimalarial target candidates.

## Author summary

Malaria is a serious infectious disease caused by parasites of the genus *Plasmodium*. The recommended treatment is a combination of antimalarial drugs. However, the rise of parasites resistant to the current antimalarial drugs means that new therapeutics are continually required. To meet this challenge, we developed and applied models using deep learning, a powerful artificial intelligence method, supported by experimental validation to identify new drug candidates against malaria. We used the developed computational models to prioritize novel, active, and nontoxic compounds from virtual chemical libraries for experimental evaluation. Then, the predicted antimalarial compounds were experimentally validated in assays on *Plasmodium falciparum* culture. This allowed us to discover two new potential antimalarial candidates. The use of computational approaches is an attractive route to expedite the discovery of new therapeutics, especially to infectious tropical diseases, as it can reduce time and development costs. Future directions include *in vivo* studies on animal models.

## Introduction

Malaria is a serious worldwide health problem that affects 216 million people, killing over 445,000 patients annually, especially children younger than five-years-old and pregnant women in Sub-Saharan Africa [1]. The disease is transmitted to humans through the bites of infected female *Anopheles* mosquitoes and caused by parasites of the genus *Plasmodium* [2,3]. Among them, *P*. *falciparum* is the most prevalent and dangerous species, causing the severe form of the disease, *i*.*e*., cerebral malaria [3,4].

Current control and eradication of malaria demands a multifaceted approach. The World Health Organization recommends a combination of at least two drugs with different mechanism of action. However, the efficacy of antimalarial drugs is threatened by the emergence and spread of resistant strains to all major antimalarial drugs, such as chloroquine [5], atovaquone [6], pyrimethamine [7], and sulfadoxine [8]. More recently, drug resistance has also been reported to front-line artemisinin-based combination therapies (ACTs) in the Greater Mekong Subregion and southeast Asia [9–11]. All these aspects highlight the compelling need for the development of new therapies to solve the challenges of drug resistance and treatment adherence by identifying molecules with novel mechanisms of action and activity against all known resistant parasite strains [12].

In this context, computational approaches, especially quantitative structure-activity relationships (QSAR) modeling, have had a profound impact in drug discovery [13]. Methodologically, QSAR modeling can be presented as a three-part process. Initially, a set of chemicals with experimentally-determined biological properties is converted into molecular descriptors (independent variables). Then, statistical methods are employed to establish relationships between descriptors and the biological properties (dependent variable) [14,15]. Early statistical methods used in QSAR applications were linear regression models [16–18], but these were quickly supplanted by Bayesian neural networks [19,20], followed by Support Vector Machines [21] and Random Forests [22–24]. Once statistically validated using appropriate metrics, the generated model represents a helpful tool for the virtual screening (VS) of new chemicals with desired biological properties.

The availability of large datasets of chemical compounds with at least one biological property measured [25,26], associated with thousands of molecular descriptors paired with the popularization of *in silico* approaches resulted in the widespread use of QSAR for a diverse array of biological properties relevant to drug discovery [27–32]. However, dealing with big datasets has posed a challenge to model biological properties using classical machine learning algorithms [33,34]. To address this issue, deep learning methods (deep neural networks) have been presented as a practical solution [35,36]. Deep learning is particularly well-suited for QSAR modeling because it possesses multiple hidden layers capable of computing adaptive non-linear features that increasingly capture complex data patterns with each iterative additional layer, which makes this approach useful for tackling more complex chemical data [37,38].

**Fig 1:**
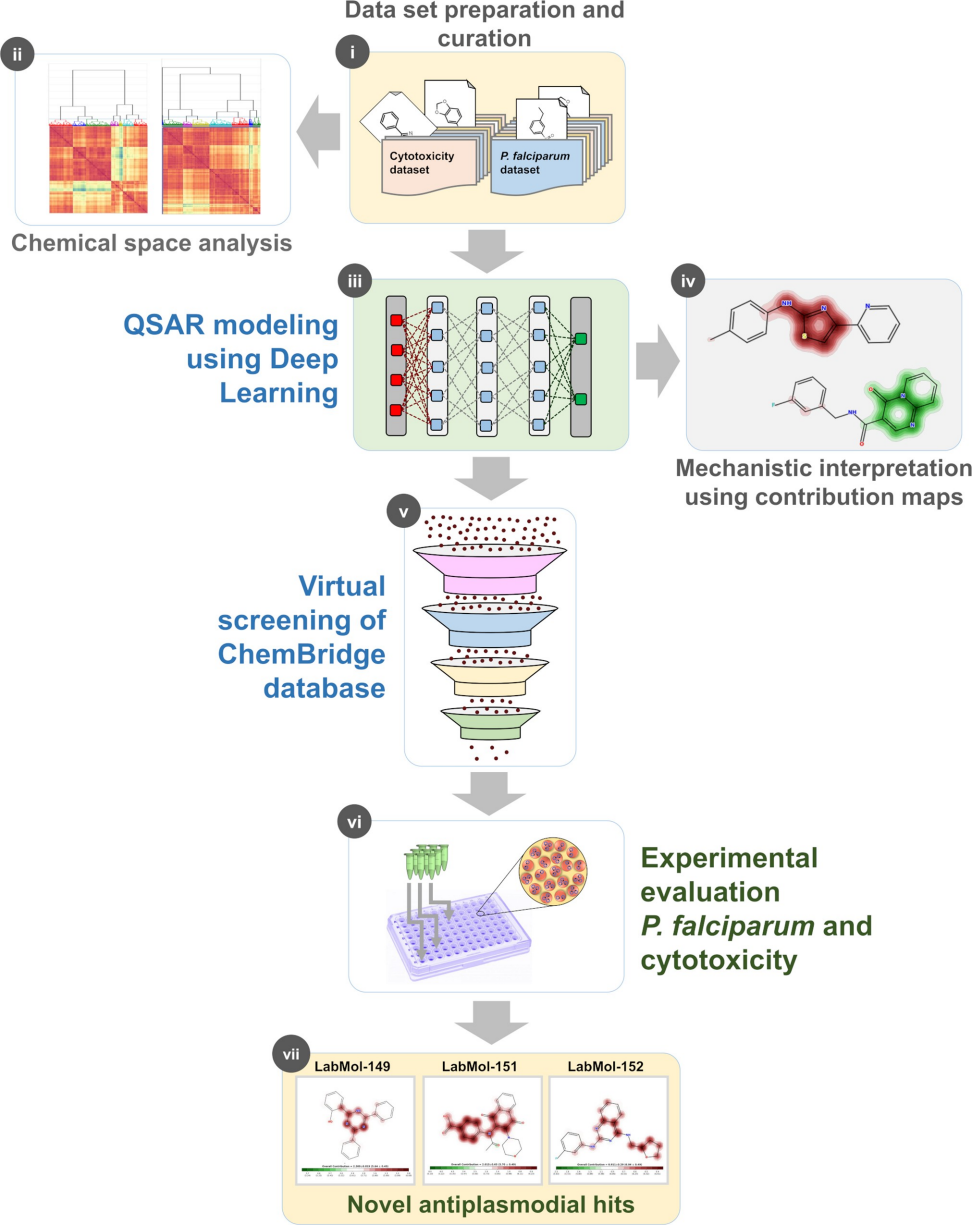
Study design. (i) dataset collection, curation, and integration of molecules with activity against *P*. *falciparum* and cytotoxicity in fibroblasts; (ii) chemical space analysis; (iii) development of QSAR models using deep learning; (iv) mechanistic interpretation of models; (v) VS of ChemBridge chemical database; (vi) experimental validation of prioritized compounds; and (vii) identification of novel antiplasmodial compounds.

Recently, there have been some exciting studies implementing deep learning for *de novo* design of molecules [39–41] compounds with desired activity. Here, we developed a modeling protocol employing deep learning to build binary and continuous QSAR models based on large datasets and applied them for predicting the antiplasmodial activity and cytotoxicity of untested compounds. Then, the prioritized compounds were experimentally evaluated against asexual blood stages *P*. *falciparum* and mammalian cells. The general study design is presented in Fig 1. Briefly, we followed the following successive steps: (i) dataset collection, curation, and integration of molecules with activity against *P*. *falciparum* and cytotoxicity in fibroblasts; (ii) chemical space analysis of curated datasets; (iii) development of both binary and continuous QSAR models using deep learning; (iv) mechanistic interpretation of continuous models to provide structural and biological insights useful for design of new antiplasmodial compounds; (v) VS of ChemBridge chemical database (∼500,000 compounds); (vi) experimental validation of prioritized compounds on asexual blood stages of *P*. *falciparum* (sensitive and multi-drug resistant strains) and mammalian cells; and (vii) identification of novel antiplasmodial compounds.

## Results

### Chemical space analysis

**Fig 2:**
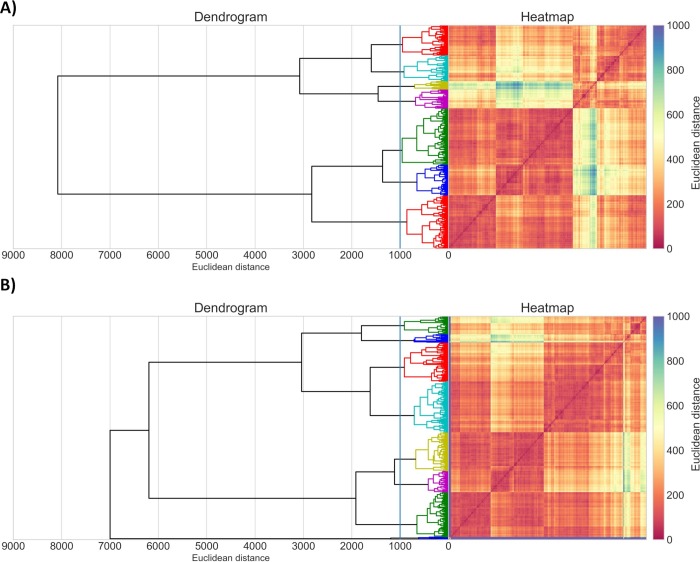
**Cluster analysis of A) 1,162 compounds from *P*. *falciparum* dataset and B) 1,270 compounds from cytotoxicity dataset.** Dendrogram and heatmap of the distance matrix are both colored according to structural similarity (orange/red = similar; blue/violet = dissimilar). The x- and y-axis labels of the heatmap represent compounds.

Initially, an activity threshold of 1 μM based on half maximal effective concentration (EC_50_) against *P*. *falciparum* was defined for discrimination between active and inactive compounds previously tested against asexual blood stages of *P*. *falciparum*. In addition, a threshold of 10 μM based on half-maximal cytotoxic concentration (CC_50_) for the NIH/3T3 cells was defined for discrimination between toxic and nontoxic compounds [42]. The analysis of chemical space was performed by using the curated datasets (see Materials and Methods) for erythrocytic stages of *P*. *falciparum* 3D7 strain (chloroquine sensitive) dataset containing 1,162 compounds (*P*. *falciparum* dataset) and cytotoxicity dataset tested against mouse embryonic fibroblasts (NIH/3T3 cell line) containing 1,270 compounds (cytotoxicity dataset). This analysis has been performed by clustering both datasets separately, which revealed that both are very structurally dissimilar, containing smaller clusters of similar compounds (Fig 2).

**Fig 3:**
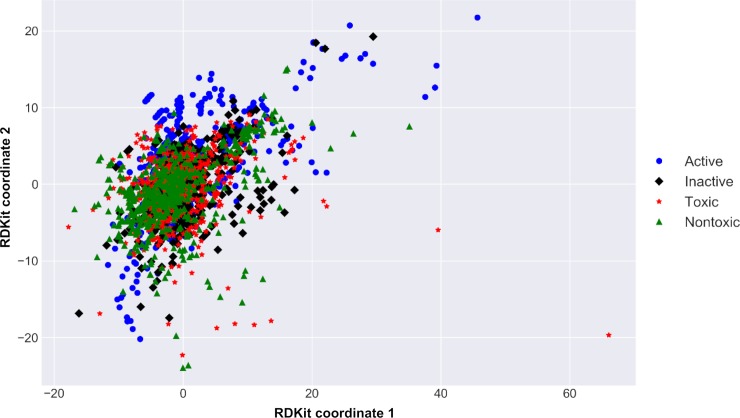
Chemical space of investigated compounds. The plot was obtained using barycentric coordinates from 2D RDKit descriptors showing active (blue dots) and inactive (black diamonds) compounds of *P*. *falciparum* dataset and toxic (red stars) and nontoxic (green triangles) from cytotoxicity dataset.

Although the datasets are structurally diverse, they share the same regions of chemical space. When analyzing the chemical space of both datasets together, by protting the two-dimensional barcentric coordinates [43] (Fig 3, see Materials and Methods for details), one can see that most of the active and inactive compounds from *P*. *falciparum* dataset overlap within the same regions of chemical space of toxic and nontoxic compounds from the cytotoxicity dataset. This analysis reveals that multiple compounds active against *P*. *falciparum* in the erythrocytic stage are potentially toxic in mouse embryonic fibroblasts. For this reason, we developed predictive computational models for both biological properties in order to select only compounds predicted as active for *P*. *falciparum* and nontoxic for mammalian cells.

### Performance of binary QSAR models

Binary QSAR models were built to distinguish active *vs*. inactive compounds for *P*. *falciparum* and toxic *vs*. nontoxic compounds for NIH/3T3 cells. According to the statistical results of a 5-fold external cross-validation procedure (see Materials and Methods), the combination of Morgan and FeatMorgan fingerprints (radius 2: FeatMorgan_2, Morgan_2; radius 4: FeatMorgan_4, Morgan_4) with deep learning (see Materials and Methods for details) led to predictive binary QSAR models. Statistical characteristics of developed QSAR models estimated by 5-fold external cross-validation are reported in Table 1. Briefly, correct classification rate (CCR) values were ranging between 0.82–0.87; sensitivity (SE)– 0.82–0.87; specificity (SP)– 0.82–0.87, and a coverage– 0.77–0.87. Table 1 shows the detailed performances of the binary QSAR models. The model built using Morgan_2 demonstrated the best performance among all other models developed for *P*. *falciparum* (CCR = 0.84; SE = 0.82; SP = 0.86; and PPV = 0.86). On the other hand, the best model developed for prediction of cytotoxicity for mammalian fibroblasts was built using FeatMorgan_4 (CCR = 0.87; SE = 0.87; and SP = 0.87).

**Table 1:** Summarized statistical characteristics of binary QSAR models.

Model	CCR	SE	SP	PPV	NPV	Coverage
***P*. *falciparum***						
FeatMorgan_2	0.84	0.82	0.86	0.85	0.83	0.80
FeatMorgan_4	0.82	0.82	0.83	0.83	0.83	0.78
Morgan_2	0.84	0.82	0.86	0.86	0.83	0.80
Morgan_4	0.84	0.83	0.85	0.85	0.83	0.77
**Cytotoxicity**						
FeatMorgan_2	0.84	0.85	0.84	0.84	0.85	0.80
FeatMorgan_4	0.87	0.87	0.87	0.87	0.87	0.85
Morgan_2	0.84	0.86	0.83	0.83	0.85	0.79
Morgan_4	0.84	0.84	0.85	0.85	0.84	0.87

CCR: correct classification rate; SE: sensitivity; SP: specificity; PPV: positive predictive value; and NPV: negative predictive value; Coverage: percentage of test set compounds within the applicability domain.

### Performance of continuous QSAR models

We have developed continuous QSAR models aiming to predict negative logarithmic units of EC_50_ values (pEC_50_) against *P*. *falciparum* and CC_50_ values (pCC_50_) against NIH/3T3 cell line. According to the statistical results of a 5-fold external cross-validation procedure, the combination of Morgan and FeatMorgan fingerprints (radius 2: FeatMorgan_2, Morgan_2) with deep learning led to statistically predictive models (Table 2), with predictive squared correlation coefficient for the test set ($Q_{ext}^{2}$) values ranging between 0.70–0.88, root mean square error of cross-validation (RMSECV) of 0.44–0.55, mean absolute error (MAE) of 0.31–0.43, and coverage of 0.79–0.81. The model built using Morgan_2 demonstrated the best performance among all other models developed for *P*. *falciparum* ($Q_{ext}^{2}$ = 0.88, RMSECV = 0.49, and MAE = 0.43). On the other hand, the best model developed for prediction of cytotoxicity for mammalian fibroblasts was built using FeatMorgan_2 ($R_{ext}^{2}$ = 0.74, RMSECV = 0.44, and MAE = 0.31).

**Table 2:** Statistical characteristics of developed continuous QSAR models.

Model	$R_{ext}^{2}$	RMSECV	MAE	$Q_{ext}^{2}$	Coverage
***P*. *falciparum***					
Morgan_2	0.88	0.49	0.43	0.88	0.79
FeatMorgan_2	0.71	0.55	0.40	0.72	0.81
**Cytotoxicity**					
Morgan_2	0.70	0.51	0.38	0.70	0.79
FeatMorgan_2	0.73	0.44	0.31	0.74	0.79

*R*^2^: correlation coefficient; RMSECV: root mean square error of cross-validation; MAE: mean absolute error; $Q_{ext}^{2}$: predictive squared correlation coefficient for the test set; Coverage: percentage of test set compounds within the applicability domain (AD).

### Model interpretation

**Fig 4:**
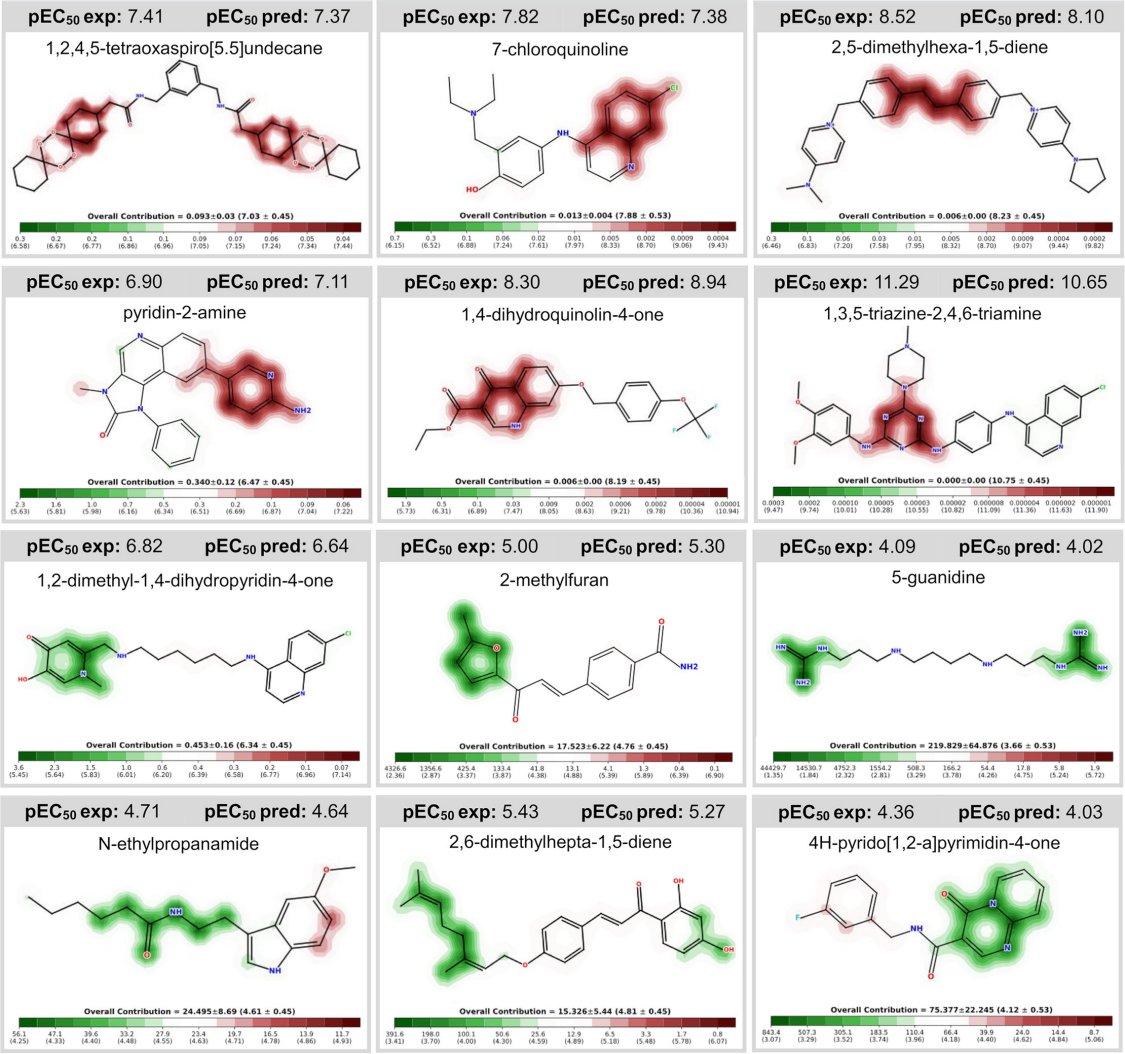
Predicted influence of structural fragments on the antiplasmodial activity. Compounds experimentally tested in P. falciparum assay, extracted from the literature and used to build/validate our models. Fragments increasing the activity are colored in red; structural moieties decreasing the activity are highlighted in green; indifferent fragments are not highlighted. pEC_50_ exp = pEC_50_ experimental; pEC_50_ pred = pEC_50_ predicted.

To provide a mechanistic interpretation and shed some light from the structural and biological data used to build the continuous QSAR models, we plotted the predicted feature (fingerprint) importance to visualize how the fragments contributed for the antiplasmodial activity and the cytotoxicity (Fig 4 and S1 Fig). According to our results, atoms or fragments promoting positive contribution for the antiplasmodial activity are highlighted in red, while structural moieties decreasing the activity are highlighted in green.

By analyzing the contribution maps generated for the *P*. *falciparum* dataset, we identified six major fragments with favorable contribution for antiplasmodial activity. Examples of favorable fragments are as follows: 1,2,4,5-tetraoxaspiro[5.5]undecane; 7-chloroquinoline; 2,5-dimethylhexa-1,5-diene; pyridin-2-amine; 1,4-dihydroquinolin-4-one; and 1,3,5-triazine-2,4,6-triamine. We also identified six fragments with unfavorable contribution for antiplasmodial activity, such as: 1,2-dimethyl-1,4-dihydropyridin-4-one; 2-methylfuran; 5-guanidine; N-ethylpropanamide; 2,6-dimethylhepta-1,5-diene; and 4H-pyrido[1,2-a]pyrimidin-4-one. Moreover, we also calculated the predicted influence of structural fragments on the cytotoxicity. A summarized list of atoms or fragments with favorable and unfavorable contribution for cytotoxicity on mammalian fibroblasts is available in S1 Fig. The structural and biological information provided by the QSAR models developed using deep learning could be useful for designing or optimizing potent and selective antiplasmodial compounds by replacing unfavorable fragments by favorable fragments, assuming true independence of physicochemical effects.

### Virtual screening

**Fig 5:**
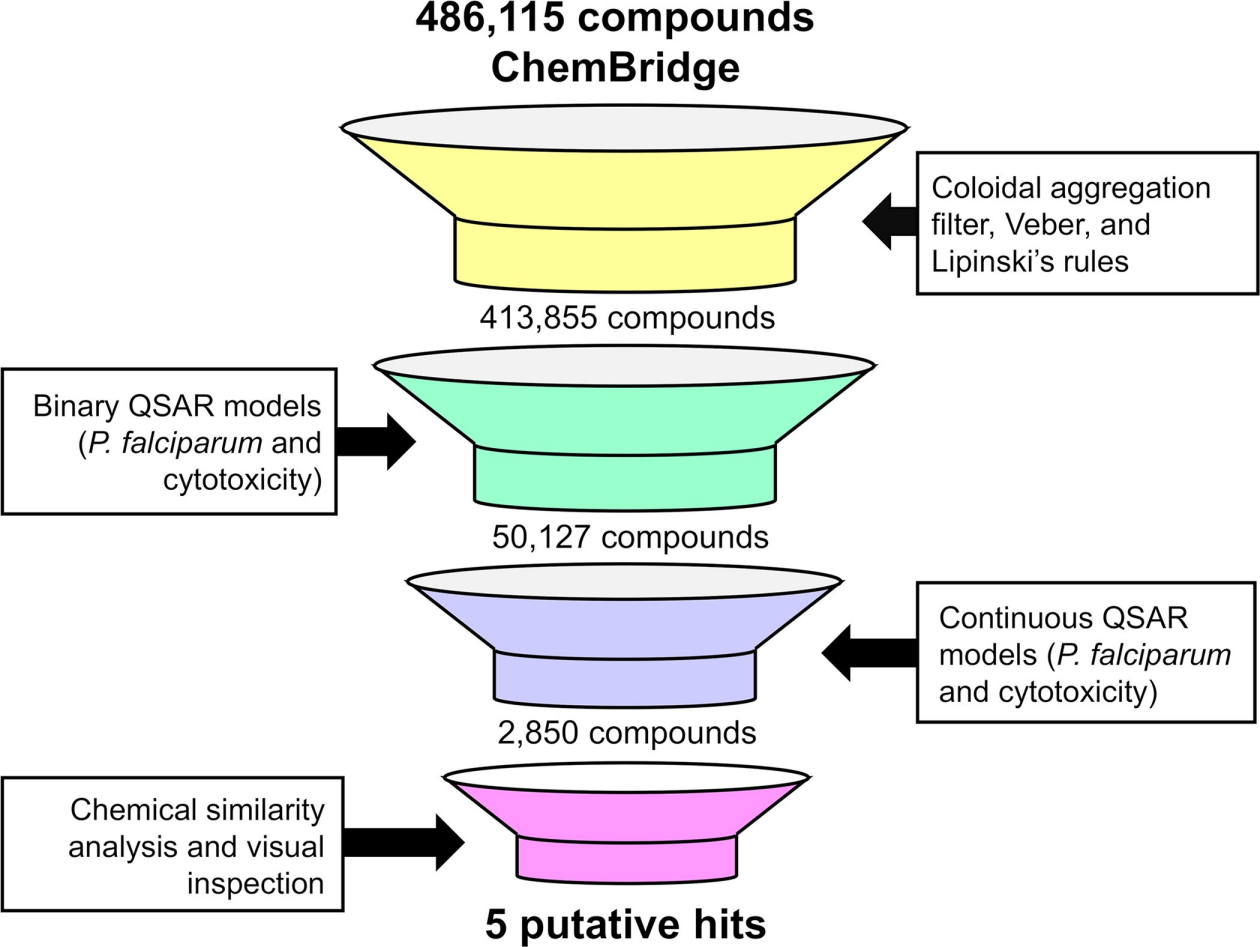
Virtual screening workflow used for identifying new compounds active against *P*. *falciparum* and non-toxic to mammalian cells. Veber and Lipinski rules were used to prioritize candidate compounds with good oral bioavailability, to ensure that the compound has basic properties of active drugs; colloidal aggregation tool was used to filter out molecules that are known to aggregate in experimental assays; chemical similarity analysis and visual inspection were performed to select a subset of structurally diverse candidate compounds.

**Fig 6:**
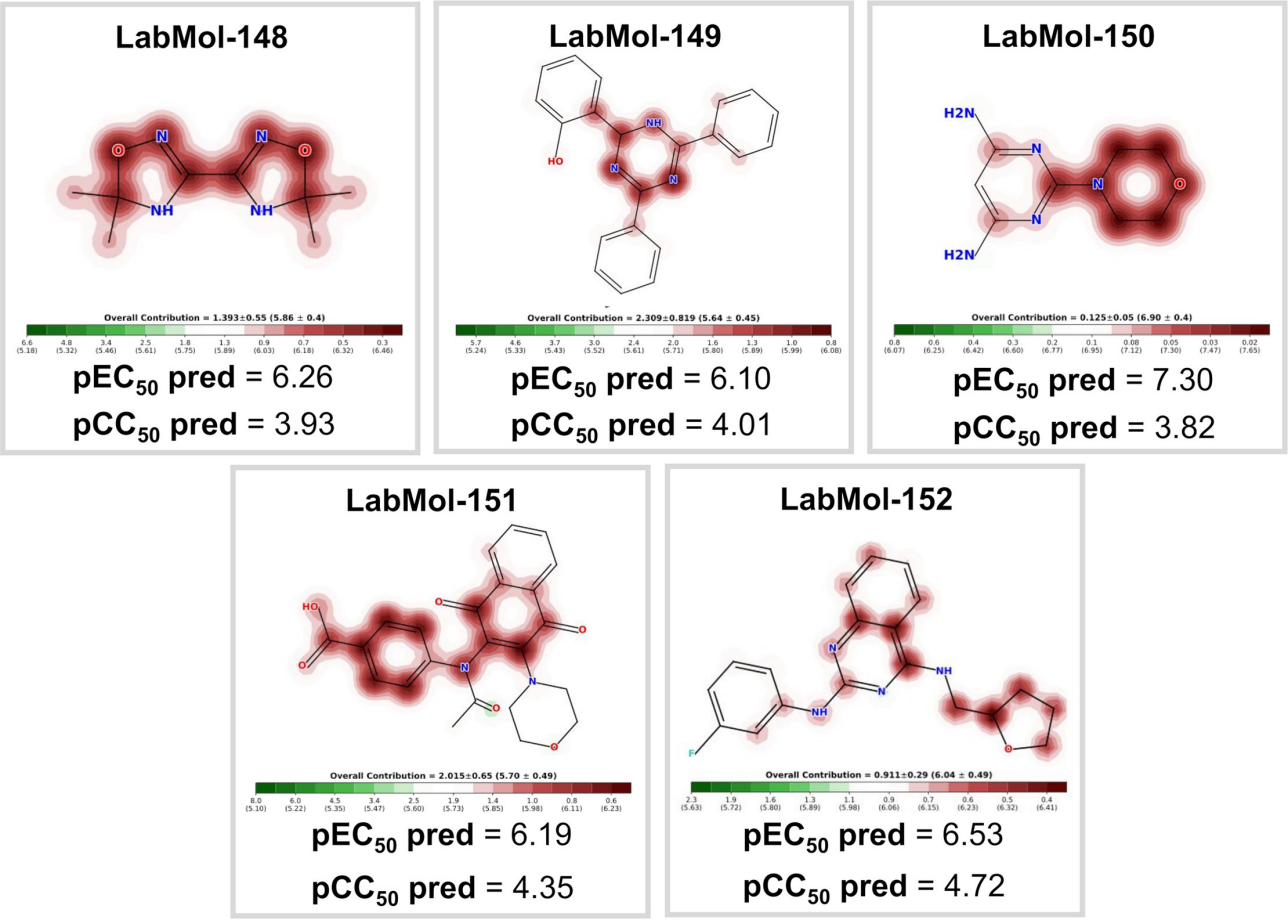
Five computationally-determined candidate compounds prioritized for further experimental evaluation. Atoms or fragments promoting positive contribution for the antiplasmodial activity are highlighted in red.

The virtual screening (VS) was carried out following the workflow presented in Fig 5. Initially, 486,115 compounds available on EXPRESS-Pick collection of ChemBridge were downloaded and standardized for VS. Then, drug-likeness filters (Veber [44] and Lipinski’s rules [45]) were applied to prioritize molecules with good oral bioavailability, to ensure that the compound has basic properties of active drugs. In parallel, colloidal aggregation tool was used to filter out molecules that are known to aggregate in experimental assays [46,47] After these steps, 72,260 compounds were excluded. Afterwards, the remaining compounds were submitted to conservative binary and continuous QSAR models for prediction of the activity against blood stages of *P*. *falciparum* and cytotoxicity against mammalian cells. The final selection of candidate compounds can be summarized as follows: (i) the compounds predicted as active and non-cytotoxic by the binary QSAR models; (ii) compounds with pEC_50_ ≥6.00 (*i*.*e*., EC_50_ ≤1 μM for *P*. *falciparum*) and pCC_50_ <5.00 (*i*.*e*., CC_50_ >10 μM for mammalian cells) predicted by the continuous QSAR models; (iii) and compounds inside the applicability domain (AD) of the QSAR models. The combination of binary and continuous QSAR models was implemented to increase success rates in virtual screening campaign. In addition, the AD was determined in order to set “reliable” and “unreliable” predictions [48,49]. The predictions were considered reliable when they were within the chemical space used for training the models. Finally, we performed a chemical similarity analysis to select a subset of structurally diverse compounds. At the end of this process, five candidate compounds were selected for biological evaluation (Fig 6).

### Experimental validation

The five candidate compounds were evaluated *in vitro* against asexual blood stages of *P*. *falciparum* sensitive (3D7), and multi-drug-resistant (W2) strains. The EC_50_ for each compound (Table 3) indicate that two compounds, 2-(4,6-diphenyl-1,2-dihydro-1,3,5-triazin-2-yl)phenol (LabMol-149), 4-{N-[3-(morpholin-4-yl)-1,4-dioxo-1,4-dihydronaphthalen-2-yl]acetamido}benzoic acid (LabMol-151) and N2-(3-fluorophenyl)-N4-[(oxolan-2-yl)methyl]quinazoline-2,4-diamine (LabMol-152), were potent at inhibiting the parasite growth, showing activities in submicromolar and low nanomolar range against both 3D7 and W2 strains. Moreover, the compound LabMol-152 (EC_50_ = 0.049 μM and 0.078 μM for 3D7 and W2, respectively) showed efficacy in the same range of activity of the reference drugs, chloroquine (EC_50_ = 0.011 μM) and pyrimethamine (EC_50_ = 0.037 μM). The candidate compounds were also evaluated for their cytotoxicity against fibroblast-like cell lines derived from monkey kidney (COS-7 cells). With respect to selectivity, LabMol-149 and LabMol-152 showed the most promising results (selectivity index, SI, ranging between 71.4–340.8, Table 3).

**Table 3:** *In vitro* evaluation of selected compounds against asexual blood stage of *P*. *falciparum* 3D7 and W2 strains, cytotoxicity on mammalian cells (COS7), and selectivity indices.

Compound	EC_50_ (*Pf*3D7) (μM)	EC_50_ (*Pf*W2) (μM)	CC_50_ (COS-7) (μM)	SI^a^	SI^b^
LabMol-148	>40	>40	>200	−	−
LabMol-149	1.450 ± 0.524	0.509 ± 0.177	>100	>71.4	>196
LabMol-150	>40	>40	>200	−	−
LabMol-151	1.911 ± 0.292	1.616 ± 0.321	140.8 ± 11.2	73.7	87.1
LabMol-152	0.049 ± 0.029	0.078 ± 0.014	16.7 ± 10.3	340.8	214.1
Chloroquine	0.011 ± 0.001	0.173 ± 0.020	>50	4,545	289
Pyrimethamine	0.037 ± 0.007	18.240 ± 4.537	>100	2,702	5.5

EC_50_: half maximal effective concentration on 3D7 and W2 *Plasmodium falciparum* strains; CC_50_: half maximal cytotoxic concentration on COS7 cells; COS7: fibroblast-like cells derived from monkey kidney tissue; SI^a^: Selectivity index calculated by COS7 CC_50_/3D7 EC_50_; and SI^b^: Selectivity index calculated by COS7 CC_50_/W2 EC_50_; The data are expressed as mean ± SD of three independent assays. Dashed SI values means that SI could be calculated because compounds did not show activity even at highest concentrations used in the assay.

## Discussion

In this work, we used a deep learning technique to obtain both binary and continuous QSAR models to predict the antiplasmodial activity and cytotoxicity of untested compounds. Models were developed following the best practices of QSAR modeling [14,15], which are fully compliant to Organization for Economic Co-operation and Development (OECD) guidance [50], such as (*i*) a defined endpoint (biological properties in our case), (*ii*) an unambiguous algorithm, (*iii*) a defined applicability domain (AD), (*iv*) appropriate measures of goodness-of-fit, robustness, and predictivity, and (*v*) mechanistic interpretation, if possible [50].

Our study follows the most recent tendencies in the usage of deep learning to probe chemical space of drug-like molecules [38,51]. As a result, we have obtained predictive QSAR models, with CCR values ranging between 0.82–0.87 (binary models) and $Q_{ext}^{2}$ values ranging between 0.70–0.88 (continuous models). These results suggest that deep learning can be efficiently used to rationalize the identification of potent and selective antiplasmodial compounds in early stages of drug discovery. In addition, analysis of chemical space of *P*. *falciparum* and cytotoxicity dataset revealed that the compounds active against *P*. *falciparum* share the same chemical space of many compounds that are toxic in mouse embryonic fibroblasts.

**Fig 7:**
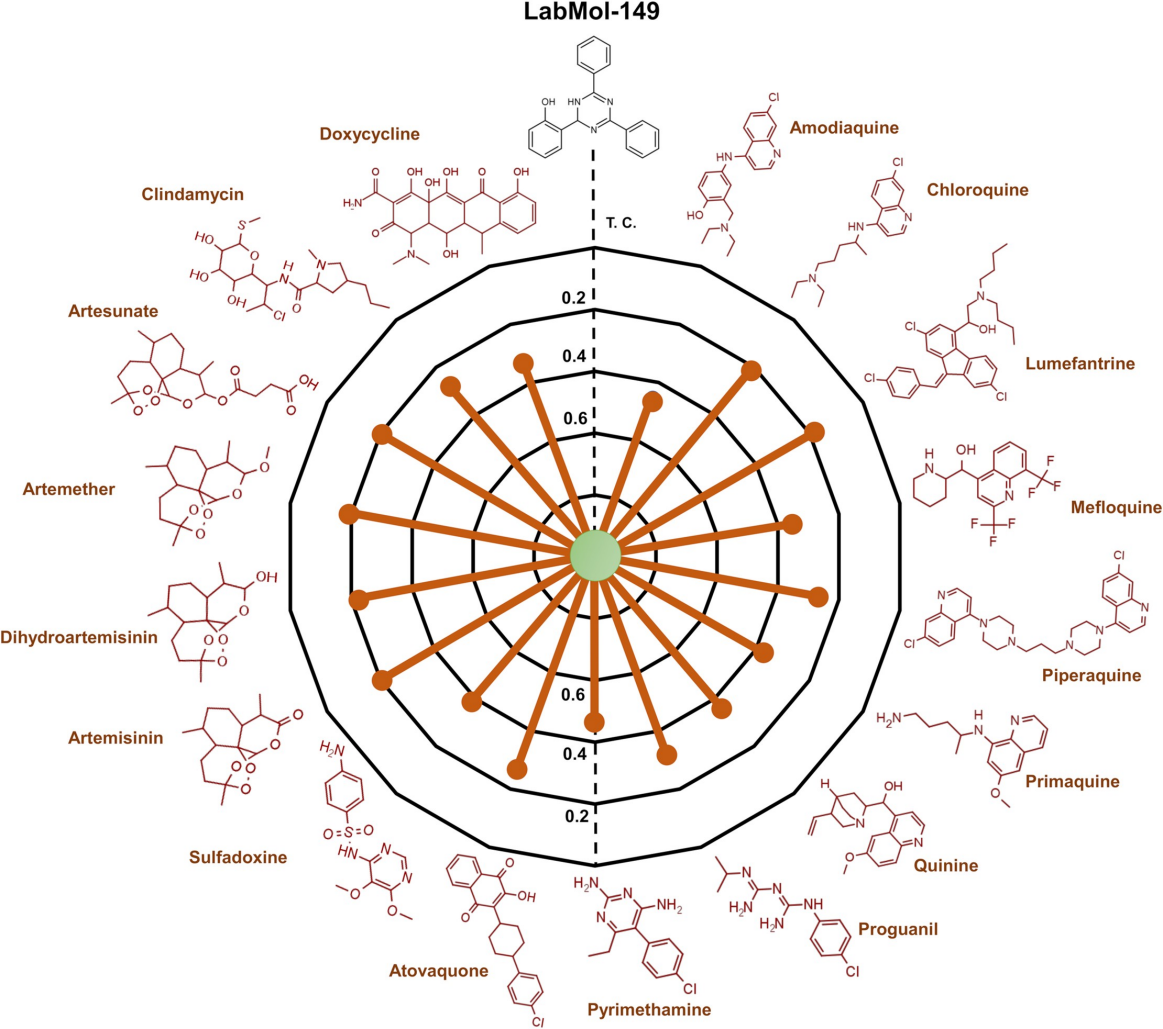
Radial plot showing the similarities of LabMol-149 to known anti-malarial drugs (red). The similarity was accessed by the Tanimoto coefficient (*Tc*) and MACCS structural key fingerprints.

By applying the workflow presented on Fig 5, we were able prioritize five new compounds for further experimental testing *in vitro* against sensitive (3D7) and multidrug-resistant (W2) strains of *P*. *falciparum*. Two compounds (LabMol-149 and LabMol-151) showed activity at submicromolar concentrations against asexual blood stages and low cytotoxicity in mammalian cells. More remarkable, the compound LabMol-152 showed efficacy in the same range of activity as the reference drug pyrimethamine against 3D7 strain (EC_50_ = 0.049 and 0.037 μM, respectively). Drug resistance in *Plasmodium* spp. is a complex daunting issue, and there is an opinion that resistant parasite strains will always emerge [12]. Although not fully understood, clinical resistance is probably due to high parasite genetic diversity and the misuse of therapeutics. The compounds tested here present chemical scaffolds dissimilar from current antimalarial drugs, according to the Tanimoto coefficient calculated using MACCS structural keys descriptors (Fig 7, S2 and S3 Figs, Supporting Information). Furthermore, no compound showed cross-resistance with a *P*. *falciparum* multidrug-resistant strain, thus indicating new mechanisms of action and potentially representing new weapons in our arsenal of antimalarials. However, parasite resistance to new compounds seems more likely to be a problem by *de novo* acquisition than pre-existing resistance [52]. Thus, how quickly resistance against our compounds would occur is an interesting question and needs to be better elucidated in future studies.

To summarize, the approach developed in this study allowed us to discover three new chemicals belonging to three different structural families (triazines, naphtoquinones, and quinazolines), which are promising starting points for developing of potential next generation antimalarial agents. Moreover, two of these compounds (LabMol-149 and LabMol-152) completely satisfy the guidelines and criteria for discovery of new antimalarial drugs, *i*.*e*., activity at low nanomolar concentrations (EC_50_ <500nM) against sensitive and multiple resistant strains of *Plasmodium* spp. and SI greater than 10 folds [12,42]. Future directions include structural optimization of potency and selectivity, determination of the stage in the asexual life cycle of *P*. *falciparum* where these compounds seem to act, as well as *in vivo* assays.

## Materials and methods

### Computational

#### Datasets

In this study, deep learning algorithms were explored to build binary and continuous QSAR models using two datasets extracted from the ChEMBL database (https://www.ebi.ac.uk/chembl/) [25]. A brief description of the datasets is presented below.

- *P*. *falciparum* (Target ID: CHEMBL2366922): 1,757 compounds with EC_50_ data for asexual blood stages of *P*. *falciparum* 3D7 strain (chloroquine sensitive). Based on a threshold of 1 μM, it consisted of 1,058 active compounds with EC_50_ ≤ 1 μM, and 699 inactive compounds (EC_50_ > 1 μM).
- Cytotoxicity (Target ID: CHEMBL614822): 2,061 compounds with CC_50_ data for mouse embryonic fibroblasts (NIH/3T3 cell line). Based on a threshold of 10 μM, it consisted of 773 toxic compounds with CC_50_ ≤ 10 μM, and 1,288 nontoxic compounds (CC_50_ > 10 μM).

#### Data curation

All chemical structures and correspondent biological information were carefully standardized using Standardizer v.16.9.5.0 (ChemAxon, Budapest, Hungary, http://www.chemaxon.com) according to the protocols proposed by Fourches and colleagues [53–55]. Briefly, explicit hydrogens were added, whereas polymers, salts, metals, organometallic compounds, and mixtures were removed. In addition, specific chemotypes such as aromatic rings and nitro groups were normalized. Furthermore, we performed the analysis and exclusion of duplicates. Different criteria were adopted, as follows:

- *Binary QSAR models*: (i) if duplicates presented discordance in biological activity, both entries would be excluded; and (ii) if the reported outcomes of the duplicates were the same, one entry would be retained in the dataset and the other excluded. This analysis showed high concordance between duplicate records for *P*. *falciparum* dataset (92%), and cytotoxicity dataset (100%), revealing the high quality of these datasets. Considering the different size of classes in *P*. *falciparum* dataset (789 actives and 581 inactives), and cytotoxicity dataset (635 toxic compounds and 933 nontoxic compounds), the curated datasets were balanced using a linear under-sampling approach (i.e., reducing the size of the majority class) [29]. The under-sampling strategy used here retains most of the representative molecules of the majority class in balanced dataset, ensuring the structural diversity of original chemical space [29]. Initially, the Euclidean distances between each compound in majority class and whole set of minority class are measured using *k*-Nearest Neighbor (*k*-NN) algorithm [56]. Then, the samples on majority classes were linearly extracted over the whole set by using k-distances and used to generate balanced datasets. Finally, we obtained two under-sampled datasets with 1,162 compounds (see full *P*. *falciparum* dataset in S1 File, Support Information), and 1,270 compounds (see full cytotoxicity dataset in S2 File, Support Information).

- *Continuous QSAR models*: (i) duplicates were inspected visually, (ii) if duplicates presented discordant potencies, both entries would be excluded; and (iii) if the reported potencies were similar, an average of the values was calculated, and one entry would be retained in the dataset. Subsequently, the EC_50_ (*P*. *falciparum*) and CC_50_ (cytotoxicity) values were converted to negative logarithmic (−log) units, pEC_50_ and pCC_50_, respectively. At the end of this process, the *P*. *falciparum* dataset had 1,246 compounds (full dataset available on S3 File, Support Information) while the cytotoxicity dataset had 1,144 compounds (full dataset available on S4 File).

#### Chemical space analysis

Chemical space formed by *P*. *falciparum* and cytotoxicity datasets was analyzed by plotting the barycentric coordinates of all the structures encountered in both datasets, which were defined by the 2D RDKit descriptors. Barycentric coordinates correspond to the location of the points of a simplex (a triangle, tetrahedron, etc.) in the space, defined by the vertices [43]. In other words, the location of a chemical in a multidimensional space of 2D RDKit descriptors has been scaled to two dimensions. In this case, a simplex is defined by all the RDKit descriptors of a particular chemical substance. Barycentric coordinates were determined using Methods of Data Analysis module of HiT QSAR software [57]. In addition, both datasets were independently clustered using the Sequential Agglomerative Hierarchical Non-overlapping method [58] implemented in Python v.3.6. Briefly, a dendrogram of the parent-child relationships between clusters and a heatmap of the proximity matrix colored according to the pairwise chemical similarity between compounds. To better visualize the clusters, the distance matrix of the compounds from the two datasets were independently calculated and the compounds were clustered.

#### Molecular fingerprints

Morgan and FeatMorgan fingerprints were calculated in the open-source cheminformatics software RDKit (http://www.rdkit.org, [59]) executed on Python v.3.6 (https://www.python.org). Both fingerprints were generated with radius 2−4 and bit vector of 2,048 bits. Morgan is a type circular fingerprint built by applying the Morgan algorithm to a set of user-supplied 2D chemical structures [60,61]. The fingerprint generation process systematically records the neighborhood of each non-hydrogen atom into multiple circular layers up to a stablished radius. The radius is a dominant parameter which controls the number and the maximum size of considered atom neighborhoods, thus it controls the complexity of fragment representation. These atom-centered substructural features are interpreted as indexes of bits in a huge virtual bit string. Each position in this bit string accounts for the presence or absence of a specific fragment feature [60,61]. The Morgan captures highly specific atomic information enabling the representation of a large set of precisely defined structural features [60]. Additionally, invariants of Morgan called as FeatMorgan fingerprints can also be calculated by including functional features (*i*.*e*., hydrogen-bond donor and acceptors, aromatic, halogen, basic and acid groups) [62].

#### Deep learning

The binary and continuous QSAR models were developed using Keras (https://keras.io/), a deep learning library, and Tensorflow (www.tensorflow.org), a GPU training and CPU for prediction), as backend. Binary models were trained using previously established activity/toxicity thresholds, while continuous models were developed using pEC_50_ (*P*. *falciparum*) and pCC_50_ (cytotoxicity). The following parameters of the deep learning method were optimized prior to model training: layer type (dense), hidden layers (8), activation function (ReLU), output layer function (sigmoid), model optimizer (Adam). The “binary cross-entropy” and “mean squared error” were used as loss functions in binary and continuous QSAR modeling, respectively. The “accuracy” and “mean absolute error” were used as parameters to judge the performance of binary and continuous models, respectively. The following hyperparameters were used for further deep learning training: epochs (5, 10, 50, 100), and batch size (10, 20, 40, 60, 80, 100).

#### 5-fold external cross-validation (5FECV)

According to the best practices of QSAR modeling [15], we chose five-fold external cross-validation for the estimation of predictivity of developed models. The procedure can be described as follows: the entire dataset of compounds was randomly divided into five subsets of equal size; then one of these subsets (20% of all compounds) is set aside as an external validation set and the remaining four sets together form the modeling set (80% of the full set). This procedure was repeated five times, allowing each of the five subsets to be used as an external validation set. Models were built using the modeling set while the compounds in momentary external set (fold) were employed to evaluation of predictive performance.

#### Performance of QSAR models

The predictive performance of binary QSAR models was evaluated using sensitivity (SE), specificity (SP), correct classification rate (CCR), positive predictive value (PPV), and negative predictive value (NPV). These metrics were calculated as follows:

$$SE=\frac{TP}{TP+FN}$$ (1)

$$SP=\frac{TN}{TN+FP}$$ (2)

$$CCR=\frac{SE+SP}{2}$$ (3)

$$PPV=\frac{TP}{TP+FP}$$ (4)

$$NPV=\frac{TN}{TN+FN}$$ (5)

Here, TP and TN represent the number of true positives and true negatives, respectively, while FP and FN represent the number of false positives and false negatives, respectively.

The predictive performance of continuous QSAR models was evaluated using correlation coefficient (*R*^2^), root mean square error of cross validation (RMSECV), mean absolute error (MAE), and predictive squared correlation coefficient for the test set ($Q_{ext}^{2}$) [63]. These metrics were calculated as follows:

$$R^{2}=1-\frac{\sum_{i=1}^{n_{test}}(Y_{obs}-Y_{pred}{)}^{2}}{\sum_{i=1}^{n_{test}}(Y_{obs}-{\bar{Y}}_{train}{)}^{2}}$$ (6)

$$RMSECV=\sqrt{\frac{\sum_{i=1}^{n_{test}}(Y_{obs}-Y_{pred}{)}^{2}}{n_{test}}}$$ (7)

$$MAE=\frac{\sum_{i=1}^{n_{test}}\left|Y_{obs}-Y_{pred}\right|}{n_{test}}$$ (8)

$$Q_{ext}^{2}=1-\frac{[\sum_{i=1}^{n_{test}}(Y_{obs}-Y_{pred}{)}^{2}]/n_{test}}{[\sum_{i=1}^{n_{test}}(Y_{obs}-{\bar{Y}}_{train}{)}^{2}]/n_{train}}$$ (9)

In the above equations, *Y*_*obs*_ represents experimental pEC_50_ or pCC_50_ value, *Y*_*pred*_ represents the predicted pEC_50_ or pCC_50_ value, *n*_*train*_ and *n*_*test*_ are the number of compounds in training and test set, respectively, and ${\bar{Y}}_{train}$ is the average of experimental values of the training set.

#### Applicability domain

The AD was estimated based on the Euclidean distances among the training set of each QSAR model generated in the 5-fold external cross-validation procedure. The distance of a test set compound to its nearest neighbor in the training set was compared to the predefined AD threshold level. The prediction was considered to be less reliable if the distance was greater than the threshold level. In our study, the AD was defined as a distance threshold (D_T_) between a compound under prediction and the closest nearest neighbors in training set. The following equation was used for calculation of distance threshold [64]:

$$D_{T}=\bar{y}+Z\sigma$$ (10)

In which ӯ is the average Euclidean distance of the k nearest neighbors within the modeling set, σ is the standard deviation of these Euclidean distances, and Z is an arbitrary parameter to control the significance level. We set the default value of this parameter Z at 0.5. If the compound distance exceeded the threshold, the prediction was considered to be less trustworthy [65].

#### Mechanistic interpretation

Contribution maps were generated from continuous QSAR models to visualize the atomic and fragment contributions for antimalarial activity and cytotoxicity. Here, the "weight" of an atom was considered as predicted-potency difference obtained when the bits in the fingerprint corresponding to the atom are removed. Then, the normalized weights were used to color the atoms in a topography-like map in which green indicating a negative difference (i.e., potency increases when the bits are removed), and red indicating a positive difference in biological property.

#### Virtual screening (VS)

Developed QSAR models were used for VS of EXPRESS-Pick collection of ChemBridge Corporation (http://www.chembridge.com/) aiming to identify new potential antiplasmodial compounds, which could be potentially selective against the parasite (i.e. non-toxic to mammalian cells). Prior to screening, the database was filtered using a aggregator advisor tool to identify molecules that are known-to aggregate in experimental assays [46,47]. Subsequently, Veber [44] and Lipinski’s rules [45] were employed in screening to prioritize drug-like compounds. Then, the remaining compounds had their antiplasmodial activity and cytotoxicity against mammalian cells predicted by binary and continuous QSAR models. In addition, the structural diversity of candidate compounds was investigated using pairwise Tanimoto coefficients between compounds. Finally, the selected candidate compounds were purchased and submitted to *in vitro* experimental evaluation.

### Experimental

#### Materials

Candidate compounds were purchased from ChemBridge (San Diego-CA, USA) and resuspended in 100% DMSO. It is important to mention that all compounds had a minimum purity of 95%. The DMEM and RPMI 1640 media were purchased from Vitrocell Embriolife (Campinas-SP, Brazil). All other reagents were purchased from Sigma-Aldrich (St. Louis-MO, USA).

#### Parasite culture

The 3D7 and W2 strains were cultured in RPMI 1640 medium supplemented with 0.05 mg/mL gentamycin, 38.4 mM HEPES, 0.2% sodium bicarbonate, and 10% O^+^ human serum, as previously described in standardized protocol [66]. Then, erythrocytes were added to the culture to obtain a 5% of hematocrit, and incubated at 37°C under 5% CO_2_ atmosphere, with daily exchange of medium. The parasitemia was monitored daily in smears, stained with Giemsa. Synchronic cultures in ring stage were obtained by two consecutive treatments, at 48h intervals with a 5% solution of D-sorbitol [67].

#### Antiplasmodial assay

Parasites synchronized at the ring stage, with 0.5% parasitemia and 2% hematocrit, were distributed in the wells of a 96-well plate. The compounds were tested in triplicates using 12-point dilution series (0.019 μM– 40 μM) over 72h. Chloroquine and pyrimethamine were used as positive controls. The *in vitro* susceptibility of parasite to tested drugs was measured by SYBR Green according to Hartwig and colleagues [68]. Briefly, 100 μL of lysis buffer (20 mM Tris, 5 mM EDTA, 0,008% wt/vol saponin, 0,08% vol/vol Triton X-100 and 0.4 μL/mL of SYBR Green) were added in each well of a new black 96-well plate and 100 μL of parasite culture incubated with drugs were transferred. After homogenization, the plates were incubated for 1h in the dark. Fluorescence was measured at 490 nm excitation and 540 nm emission (CLARIOstar, Labtech BMG).

#### Cytotoxicity assay

Cytotoxicity assays used fibroblast-like cell lines derived from monkey kidney tissue (COS7 cells), grown in DMEM medium supplemented with 10% fetal bovine serum and 0.05 mg/mL gentamicin in atmosphere containing 5% CO_2_ at 37°C. Drug cytotoxicity in COS-7 cells was determined in duplicate, using 12 dilution series (0.097 μM– 200 μM). After an incubation period (72 hours), cell viability analysis was performed via the MMT reduction method (3- [4,5- dimethyl-thiazol-2-yl] -2,5-diphenyltetrazolium chloride) [69]. The optical density was determined at 570 nm (CLARIOstar, Labtech BMG) and the 50% cytotoxicity concentrations (CC_50_) was expressed as the percent viability relative to the control.

#### Statistics

The EC_50_ and CC_50_ values were calculated by plotting the Log doses *vs*. inhibition (expressed as a percentage relative to the control) in GraphPad Prism v.6 (GraphPad Software, La Jolla California USA, www.graphpad.com).

## Supporting information

S1 File Full *P*. *falciparum* dataset used to build binary QSAR models. (XLSX)

S2 File Full cytotoxicity dataset used to build binary QSAR models. (XLSX)

S3 File Full *P*. *falciparum* dataset used to build continuous QSAR models. (XLSX)

S4 File Full cytotoxicity dataset used to build continuous QSAR models. (XLSX)

S1 Fig Predicted influence of structural fragments on cytotoxicity. Fragments contributing for the cytotoxicity are colored in red, atoms or fragments decreasing the cytotoxicity are highlighted in green, and no highlighting means no influence to cytotoxicity.
(TIF)

S2 Fig Radial plot of the similarity of LabMol-151 compared to known antimalarial drugs. The similarity was calculated using Tanimoto coefficient (Tc) and MACCS structural keys descriptors.
(TIF)

S3 Fig Radial plot of the similarity of LabMol-152 compared to known antimalarial drugs. The similarity was calculated using Tanimoto coefficient (Tc) and MACCS structural keys descriptors.
(TIF)

## References

[1] World Health Organization, WHO, Phillips MA, Burrows JN, Manyando C, van Huijsduijnen RH, et al. Malaria. In: Nature Reviews Disease Primers [Internet]. Aug 2017 [cited 3 Nov 2017] pp. 1–24. 10.1038/nrdp.2017.50 28770814

[2] Ashley EA, Pyae Phyo A, Woodrow CJ. Malaria. Lancet. 2018;391: 1608–1621. 10.1016/S0140-6736(18)30324-6 29631781

[3] Phillips MA, Burrows JN, Manyando C, van Huijsduijnen RH, Van Voorhis WC, Wells TNC. Malaria. Nat Rev Dis Prim. 2017;3: 17050 10.1038/nrdp.2017.50 28770814

[4] Wassmer SC, Grau GER. Severe malaria: what’s new on the pathogenesis front? Int J Parasitol. 2017;47: 145–152. 10.1016/j.ijpara.2016.08.002 27670365 PMC5285481

[5] Wellems TE, Plowe C V. Chloroquine‐Resistant Malaria. J Infect Dis. 2001;184: 770–776. 10.1086/322858 11517439

[6] Srivastava IK, Morrisey JM, Darrouzet E, Daldal F, Vaidya AB. Resistance mutations reveal the atovaquone-binding domain of cytochrome b in malaria parasites. Mol Microbiol. 1999;33: 704–711. 10.1046/j.1365-2958.1999.01515.x 10447880

[7] Wu Y, Kirkman LA, Wellems TE. Transformation of Plasmodium falciparum malaria parasites by homologous integration of plasmids that confer resistance to pyrimethamine. Proc Natl Acad Sci U S A. 1996;93: 1130–1134. 10.1073/pnas.93.3.1130 8577727 PMC40043

[8] Triglia T, Wang P, Sims PFG, Hyde JE, Cowman AF. Allelic exchange at the endogenous genomic locus in Plasmodium falciparum proves the role of dihydropteroate synthase in sulfadoxine-resistant malaria. EMBO J. 1998;17: 3807–3815. 10.1093/emboj/17.14.3807 9669998 PMC1170716

[9] Rogers WO, Sem R, Tero T, Chim P, Lim P, Muth S, et al Failure of artesunate-mefloquine combination therapy for uncomplicated Plasmodium falciparum malaria in southern Cambodia. Malar J. 2009;8: 10 10.1186/1475-2875-8-10 19138388 PMC2628668

[10] Ashley EA, Dhorda M, Fairhurst RM, Amaratunga C, Lim P, Suon S, et al Spread of Artemisinin Resistance in Plasmodium falciparum Malaria. N Engl J Med. 2014;371: 411–423. 10.1056/NEJMoa1314981 25075834 PMC4143591

[11] Witkowski B, Khim N, Chim P, Kim S, Ke S, Kloeung N, et al Reduced Artemisinin Susceptibility of Plasmodium falciparum Ring Stages in Western Cambodia. Antimicrob Agents Chemother. 2013;57: 914–923. 10.1128/AAC.01868-12 23208708 PMC3553720

[12] Burrows JN, Duparc S, Gutteridge WE, Hooft van Huijsduijnen R, Kaszubska W, Macintyre F, et al New developments in anti-malarial target candidate and product profiles. Malar J. 2017;16: 26 10.1186/s12936-016-1675-x 28086874 PMC5237200

[13] Schneider G. Automating drug discovery. Nat Rev Drug Discov. Nature Publishing Group; 2017;17: 97–113. 10.1038/nrd.2017.232 29242609

[14] Tropsha A. Best Practices for QSAR Model Development, Validation, and Exploitation. Mol Inform. 2010;29: 476–488. 10.1002/minf.201000061 27463326

[15] Cherkasov A, Muratov EN, Fourches D, Varnek A, Baskin II, Cronin M, et al QSAR modeling: where have you been? Where are you going to? J Med Chem. 2014;57: 4977–5010. 10.1021/jm4004285 24351051 PMC4074254

[16] Hansch C, Fujita T. p -σ-π Analysis. A Method for the Correlation of Biological Activity and Chemical Structure. J Am Chem Soc. 1964;86: 1616–1626. 10.1021/ja01062a035

[17] Cramer RD, Patterson DE, Bunce JD. Comparative Molecular Field Analysis (CoMFA). 1. Effect of Shape on Binding of Steroids to Carrier Proteins. J Am Chem Soc. 1988;110: 5959–5967. 10.1021/ja00226a005 22148765

[18] Kubinyi H, Kehrhahn OH. Quantitative structure-activity relationships. VI. Non-linear dependence of biological activity on hydrophobic character: calculation procedures for bilinear model. Arzneimittelforschung. 1978;28: 598–601. 581935

[19] Ajay Walters WP, Murcko MA. Can We Learn To Distinguish between “Drug-like” and “Nondrug-like” Molecules? J Med Chem. 1998;41: 3314–3324. 10.1021/jm970666c 9719583

[20] Burden FR, Winkler DA. Robust QSAR models using bayesian regularized neural networks. J Med Chem. 1999;42: 3183–3187. 10.1021/jm980697n 10447964

[21] Du H, Wang J, Hu Z, Yao X, Zhang X. Prediction of fungicidal activities of rice blast disease based on least-squares support vector machines and project pursuit regression. J Agric Food Chem. 2008;56: 10785–10792. 10.1021/jf8022194 18950187

[22] Svetnik V, Liaw A, Tong C, Christopher Culberson J, Sheridan RP, Feuston BP. Random Forest: A Classification and Regression Tool for Compound Classification and QSAR Modeling. J Chem Inf Comput Sci. 2003;43: 1947–1958. 10.1021/ci034160g 14632445

[23] Alves VM, Muratov E, Fourches D, Strickland J, Kleinstreuer N, Andrade CH, et al Predicting chemically-induced skin reactions. Part I: QSAR models of skin sensitization and their application to identify potentially hazardous compounds. Toxicol Appl Pharmacol. 2015;284: 262–272. 10.1016/j.taap.2014.12.014 25560674 PMC4546933

[24] Alves VM, Muratov E, Fourches D, Strickland J, Kleinstreuer N, Andrade CH, et al Predicting chemically-induced skin reactions. Part II: QSAR models of skin permeability and the relationships between skin permeability and skin sensitization. Toxicol Appl Pharmacol. Elsevier Inc.; 2015;284: 273–280. 10.1016/j.taap.2014.12.013 25560673 PMC4408226

[25] Gaulton A, Bellis LJ, Bento AP, Chambers J, Davies M, Hersey A, et al ChEMBL: a large-scale bioactivity database for drug discovery. Nucleic Acids Res. 2012;40: D1100–D1107. 10.1093/nar/gkr777 21948594 PMC3245175

[26] Wang Y, Xiao J, Suzek TO, Zhang J, Wang J, Zhou Z, et al PubChem’s BioAssay Database. Nucleic Acids Res. 2012;40: D400–D412. 10.1093/nar/gkr1132 22140110 PMC3245056

[27] Braga RC, Alves VM, Silva AC, Nascimento MN, Silva FC, Liao LM, et al Virtual screening strategies in medicinal chemistry: the state of the art and current challenges. Curr Top Med Chem. 2014;14: 1899–1912. 10.2174/1568026614666140929120749 25262801

[28] Braga RC, Alves VM, Silva MFB, Muratov E, Fourches D, Tropsha A, et al Tuning HERG out: antitarget QSAR models for drug development. Curr Top Med Chem. 2014;14: 1399–1415. 10.2174/1568026614666140506124442 24805060 PMC4593700

[29] Neves BJ, Dantas RF, Senger MR, Melo-Filho CC, Valente WCG, de Almeida ACM, et al Discovery of New Anti-Schistosomal Hits by Integration of QSAR-Based Virtual Screening and High Content Screening. J Med Chem. 2016;59: 7075–7088. 10.1021/acs.jmedchem.5b02038 27396732 PMC5844225

[30] Melo-Filho CCCC, Dantas RF, Braga RCRCRC, Neves BJBJBJ, Senger MR, Valente WCGWCG, et al QSAR-Driven Discovery of Novel Chemical Scaffolds Active against Schistosoma mansoni. J Chem Inf Model. 2016;56: 1357–1372. 10.1021/acs.jcim.6b00055 27253773 PMC5283162

[31] Lima MNN, Melo-Filho CC, Cassiano GC, Neves BJ, Alves VM, Braga RC, et al QSAR-Driven Design and Discovery of Novel Compounds With Antiplasmodial and Transmission Blocking Activities. Front Pharmacol. 2018;9: 146 10.3389/fphar.2018.00146 29559909 PMC5845645

[32] Gomes MN, Braga RC, Grzelak EM, Neves BJ, Muratov E, Ma R, et al QSAR-driven design, synthesis and discovery of potent chalcone derivatives with antitubercular activity. Eur J Med Chem. 2017;137: 126–138. 10.1016/j.ejmech.2017.05.026 28582669 PMC6031314

[33] Zhu H, Zhang J, Kim MT, Boison A, Sedykh A, Moran K. Big Data in Chemical Toxicity Research: The Use of High-Throughput Screening Assays To Identify Potential Toxicants. Chem Res Toxicol. 2014;27: 1643–1651. 10.1021/tx500145h 25195622 PMC4203392

[34] Hartung T. Making big sense from big data in toxicology by read-across. ALTEX. 2016;33: 83–93. 10.14573/altex.1603091 27032088

[35] Ramsundar B, Liu B, Wu Z, Verras A, Tudor M, Sheridan RP, et al Is Multitask Deep Learning Practical for Pharma? J Chem Inf Model. 2017;57: 2068–2076. 10.1021/acs.jcim.7b00146 28692267

[36] Ekins S. The Next Era: Deep Learning in Pharmaceutical Research. Pharm Res. Pharmaceutical Research; 2016; 2594–2603. 10.1007/s11095-016-2029-7 27599991 PMC5042864

[37] Goh GB, Hodas NO, Vishnu A. Deep learning for computational chemistry. J Comput Chem. 2017;38: 1291–1307. 10.1002/jcc.24764 28272810

[38] Ma J, Sheridan RP, Liaw A, Dahl GE, Svetnik V. Deep Neural Nets as a Method for Quantitative Structure–Activity Relationships. J Chem Inf Model. 2015;55: 263–274. 10.1021/ci500747n 25635324

[39] Popova M, Isayev O, Tropsha A. Deep reinforcement learning for de novo drug design. Sci Adv. 2018;4: eaap7885 10.1126/sciadv.aap7885 30050984 PMC6059760

[40] Gómez-Bombarelli R, Wei JN, Duvenaud D, Hernández-Lobato JM, Sánchez-Lengeling B, Sheberla D, et al Automatic Chemical Design Using a Data-Driven Continuous Representation of Molecules. ACS Cent Sci. 2018;4: 268–276. 10.1021/acscentsci.7b00572 29532027 PMC5833007

[41] Bjerrum E, Sattarov B. Improving Chemical Autoencoder Latent Space and Molecular De Novo Generation Diversity with Heteroencoders. Biomolecules. 2018;8: 131 10.3390/biom8040131 30380783 PMC6316879

[42] Katsuno K, Burrows JN, Duncan K, van Huijsduijnen RH, Kaneko T, Kita K, et al Hit and lead criteria in drug discovery for infectious diseases of the developing world. Nat Rev Drug Discov. 2015;14: 751–758. 10.1038/nrd4683 26435527

[43] Vityuk N, Voskresenskaja E, Kuz’min V. The Synergism of Methods Barycentric Coordinates and Trend-vector for Solution ―Structure-Property Tasks. Pattern Recognit Image Anal. 1999;3: 521–528.

[44] Veber DF, Johnson SR, Cheng HY, Smith BR, Ward KW, Kopple KD. Molecular properties that influence the oral bioavailability of drug candidates. J Med Chem. 2002;45: 2615–2623. 10.1021/jm020017n 12036371

[45] Lipinski CA, Lombardo F, Dominy BW, Feeney PJ. Experimental and computational approaches to estimate solubility and permeability in drug discovery and development settings. Adv Drug Deliv Rev. 1997;23: 3–25. 10.1016/S0169-409X(96)00423-1 11259830

[46] Irwin JJ, Duan D, Torosyan H, Doak AK, Ziebart KT, Sterling T, et al An Aggregation Advisor for Ligand Discovery. J Med Chem. 2015;58: 7076–87. 10.1021/acs.jmedchem.5b01105 26295373 PMC4646424

[47] Owen SC, Doak AK, Wassam P, Shoichet MS, Shoichet BK. Colloidal Aggregation Affects the Efficacy of Anticancer Drugs in Cell Culture. ACS Chem Biol. 2012;7: 1429–1435. 10.1021/cb300189b 22625864 PMC3423826

[48] Gadaleta D, Mangiatordi GF, Catto M, Carotti A, Nicolotti O. Applicability Domain for QSAR Models. Int J Quant Struct Relationships. 2016;1: 45–63. 10.4018/IJQSPR.2016010102

[49] Netzeva TI, Worth A, Aldenberg T, Benigni R, Cronin MTD, Gramatica P, et al Current status of methods for defining the applicability domain of (quantitative) structure-activity relationships. The report and recommendations of ECVAM Workshop 52. Altern Lab Anim. 2005;33: 155–73. 10.1177/026119290503300209 16180989

[50] OECD principles for the validation, for regulatory purposes, of (Quantitative) Structure-Activity Relationship models. In: Organisation for Economic Cooperation and Development [Internet]. 2004 [cited 1 Oct 2015] pp. 1–2. Available: http://www.oecd.org/chemicalsafety/risk-assessment/37849783.pdf

[51] Ghasemi F, Mehridehnavi A, Pérez-Garrido A, Pérez-Sánchez H. Neural network and deep-learning algorithms used in QSAR studies: merits and drawbacks. Drug Discov Today. 2018;23: 1784–1790. 10.1016/j.drudis.2018.06.016 29936244

[52] Corey VC, Lukens AK, Istvan ES, Lee MCS, Franco V, Magistrado P, et al A broad analysis of resistance development in the malaria parasite. Nat Commun. 2016;7: 11901 10.1038/ncomms11901 27301419 PMC4912613

[53] Fourches D, Muratov E, Tropsha A. Trust, but verify: on the importance of chemical structure curation in cheminformatics and QSAR modeling research. J Chem Inf Model. 2010;50: 1189–204. 10.1021/ci100176x 20572635 PMC2989419

[54] Fourches D, Muratov E, Tropsha A. Curation of chemogenomics data. Nat Chem Biol. Nature Publishing Group; 2015;11: 535–535. 10.1038/nchembio.1881 26196763

[55] Fourches D, Muratov E, Tropsha A. Trust, but Verify II: A Practical Guide to Chemogenomics Data Curation. J Chem Inf Model. 2016;56: 1243–1252. 10.1021/acs.jcim.6b00129 27280890 PMC5657146

[56] Altman N. An introduction to kernel and nearest-neighbor nonparametric regression. Am Stat. 1992;46: 175–185. 10.1080/00031305.1992.10475879

[57] Kuz’min VE, Artemenko AG, Muratov EN. Hierarchical QSAR technology based on the Simplex representation of molecular structure. J Comput Aided Mol Des. ELSEVIER SCIENCE BV; 2008;22: 403–21. 10.1007/s10822-008-9179-6 18253701

[58] Varnek A, Fourches D, Horvath D, Klimchuk O, Gaudin C, Vayer P, et al ISIDA—Platform for Virtual Screening Based on Fragment and Pharmacophoric Descriptors. Curr Comput Aided-Drug Des. 2008;4: 191–198. 10.2174/157340908785747465

[59] Riniker S, Landrum G a. Open-source platform to benchmark fingerprints for ligand-based virtual screening. J Cheminform. 2013;5: 26 10.1186/1758-2946-5-26 23721588 PMC3686626

[60] Rogers D, Hahn M. Extended-Connectivity Fingerprints. J Chem Inf Model. 2010;50: 742–754. 10.1021/ci100050t 20426451

[61] Morgan HL. The Generation of a Unique Machine Description for Chemical Structures-A Technique Developed at Chemical Abstracts Service. J Chem Doc. 1965;5: 107–113. 10.1021/c160017a018

[62] Gobbi A, Poppinger D. Genetic optimization of combinatorial libraries. Biotechnol Bioeng. 1998;61: 47–54. 10.1002/(sici)1097-0290(199824)61:1<47::aid-bit9>3.0.co;2-z 10099495

[63] Consonni V, Ballabio D, Todeschini R. Comments on the Definition of the Q 2 Parameter for QSAR Validation. J Chem Inf Model. 2009;49: 1669–1678. 10.1021/ci900115y 19527034

[64] Golbraikh A, Shen M, Xiao Z, Xiao Y-D, Lee K-H, Tropsha A. Rational selection of training and test sets for the development of validated QSAR models. J Comput Aided Mol Des. 2003;17: 241–253. 10.1023/a:1025386326946 13677490

[65] Tropsha A, Golbraikh A. Predictive QSAR Modeling Workflow, Model Applicability Domains, and Virtual Screening. Curr Pharm Des. 2007;13: 3494–3504. 10.2174/138161207782794257 18220786

[66] Trager W, Jensen JB. Human malaria parasites in continuous culture. Science. 1976;193: 673–5. 10.1126/science.781840 781840

[67] Lambros C, Vanderberg JP. Synchronization of Plasmodium falciparum Erythrocytic Stages in Culture. J Parasitol. 1979;65: 418 10.2307/3280287 383936

[68] Hartwig CL, Ahmed AOA, Cooper RA, Stedman TT. SYBR Green I®-Based Parasite Growth Inhibition Assay for Measurement of Antimalarial Drug Susceptibility in Plasmodium falciparum In: Moll K, Kaneko A, Scherf A, Wahlgren M, editors. Methods in Malaria Research. 6th ed Glasgow: EVIMalaR; 2013 pp. 122–129.

[69] Mosmann T. Rapid colorimetric assay for cellular growth and survival: Application to proliferation and cytotoxicity assays. J Immunol Methods. 1983;65: 55–63. 10.1016/0022-1759(83)90303-4 6606682

