# Supplementary figures and images for: Deep Learning-driven research for drug discovery: Tackling Malaria
Source: PLoS Comput Biol. 2020 Feb 18;16(2):e1007025. doi: 10.1371/journal.pcbi.1007025 (PMC7048302; doi:10.1371/journal.pcbi.1007025)

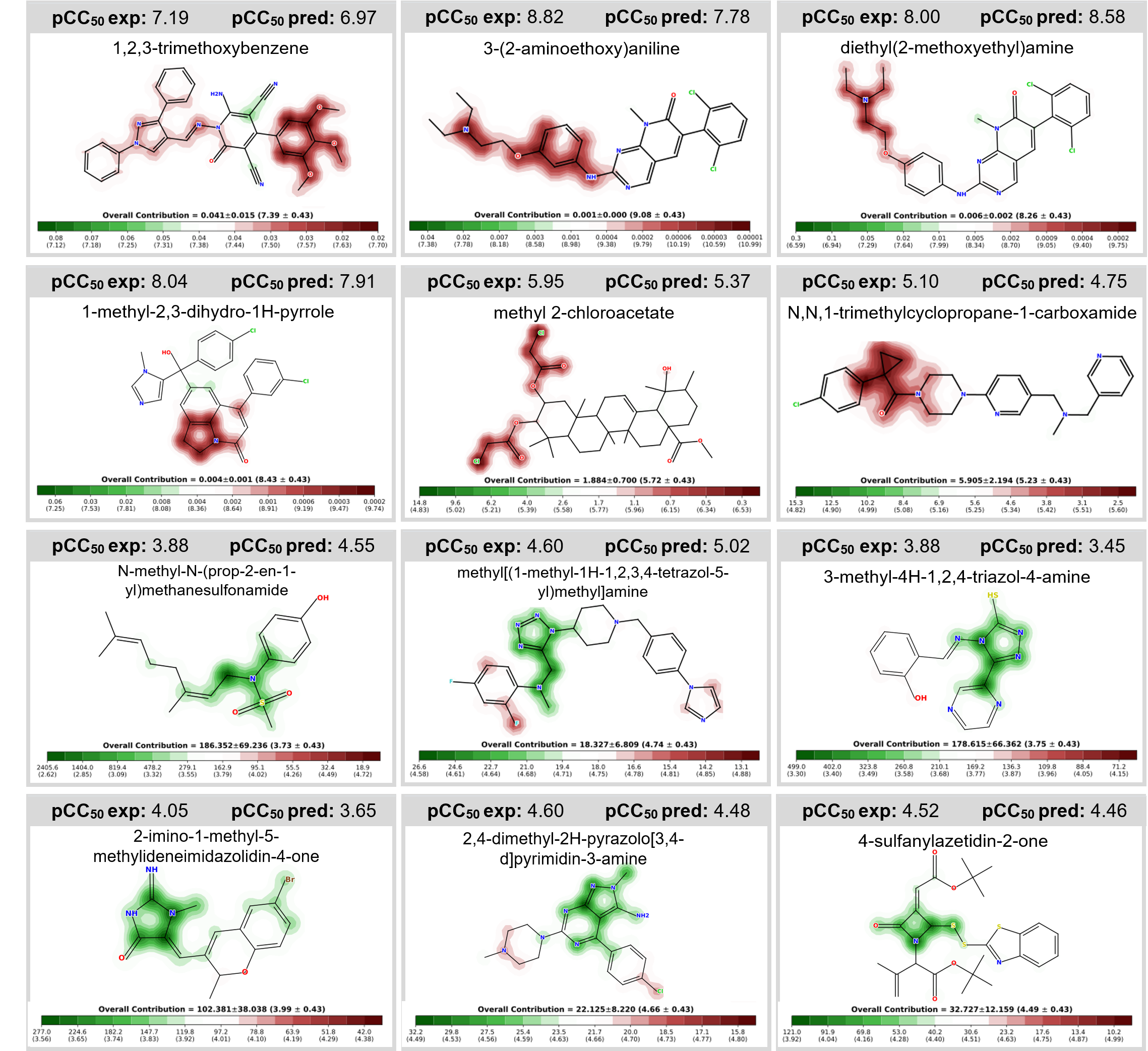

Supplement: S1 Fig — Fragments contributing for the cytotoxicity are colored in red, atoms or fragments decreasing the cytotoxicity are highlighted in green, and no highlighting means no influence to cytotoxicity. (TIF) [file pcbi.1007025.s005.tif]

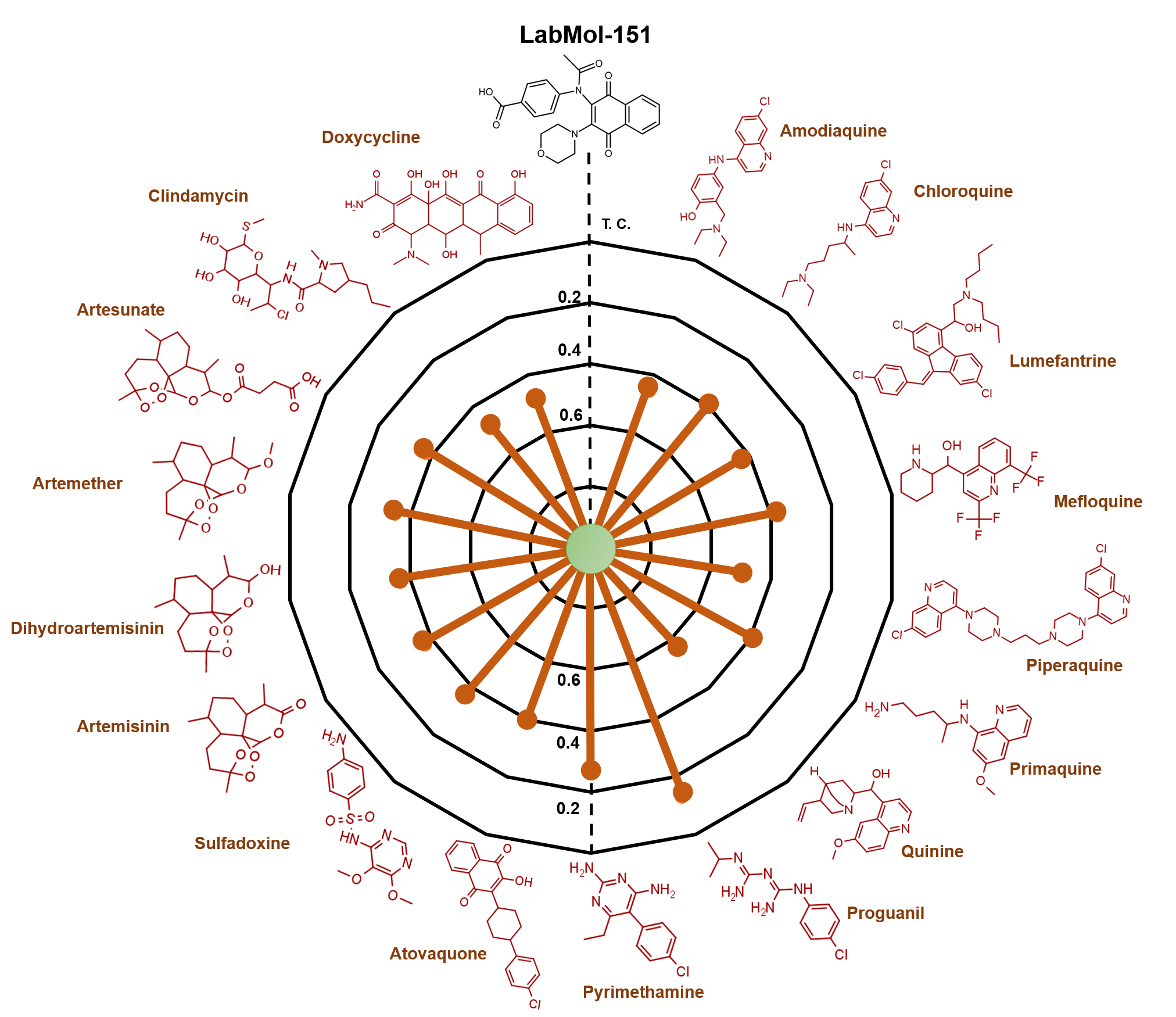

Supplement: S2 Fig — The similarity was calculated using Tanimoto coefficient (Tc) and MACCS structural keys descriptors. (TIF) [file pcbi.1007025.s006.tif]

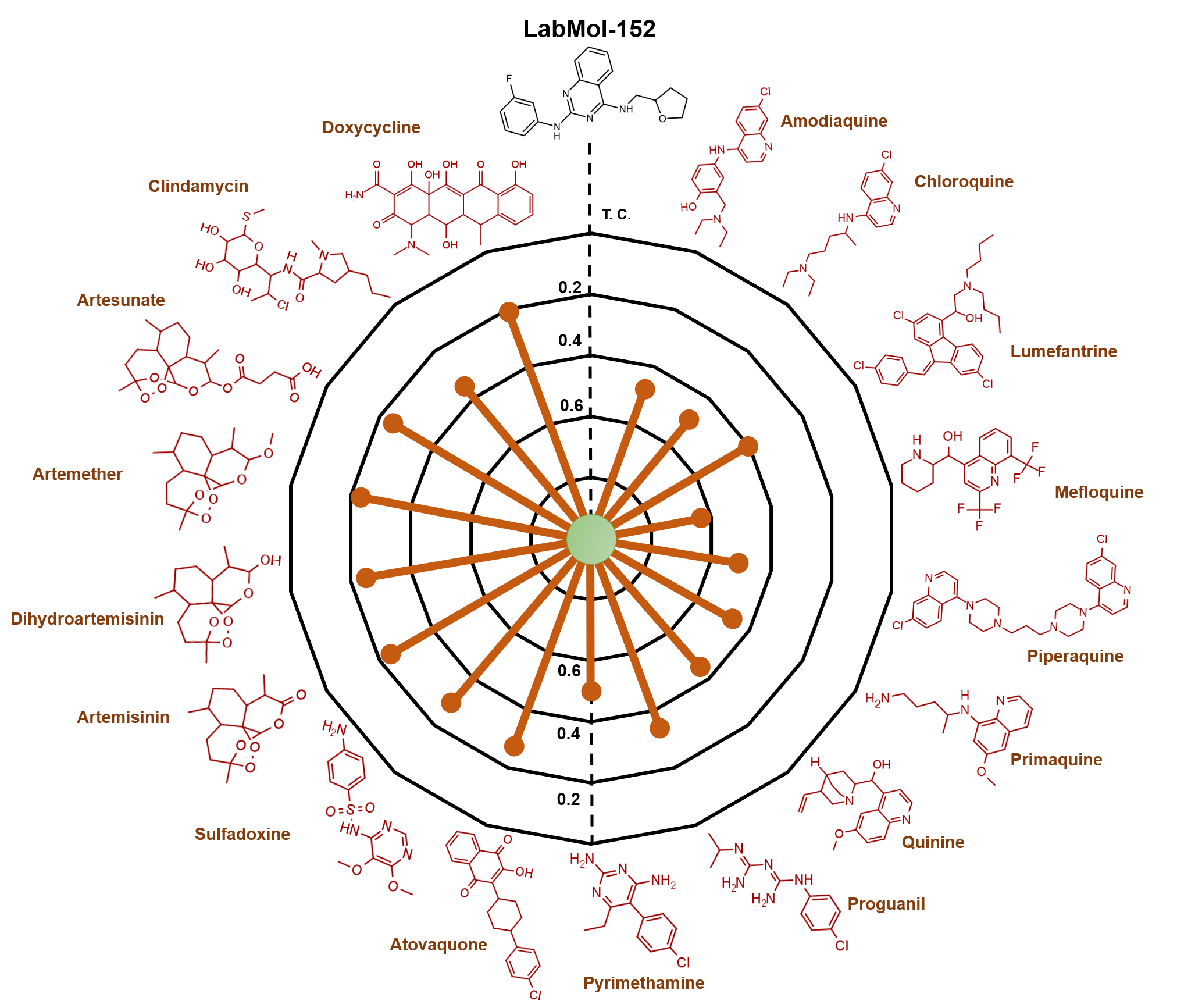

Supplement: S3 Fig — The similarity was calculated using Tanimoto coefficient (Tc) and MACCS structural keys descriptors. (TIF) [file pcbi.1007025.s007.tif]
